# Ciclesonide uptake and metabolism in human alveolar type II epithelial cells (A549)

**DOI:** 10.1186/1471-2210-7-12

**Published:** 2007-09-27

**Authors:** Takashi Nonaka, Rüdiger Nave, Nigel McCracken, Atsuko Kawashimo, Yasuhiro Katsuura

**Affiliations:** 1 Teijin Institute for Biomedical Research, Teijin Pharma Limited, 4-3-2 Asahigaoka, Hino, Tokyo 191-8512, Japan; 2 Nycomed GmbH, Byk-Gulden-Str. 2, 78467 Konstanz, Germany

## Abstract

**Background:**

Ciclesonide is a novel inhaled corticosteroid for the treatment of airway inflammation. In this study we investigated uptake and *in vitro *metabolism of ciclesonide in human alveolar type II epithelial cells (A549). Ciclesonide uptake was compared with fluticasone propionate, an inhaled corticosteroid that is not metabolized in lung tissue. A549 cells were incubated with 2 × 10^-8 ^M ciclesonide or fluticasone propionate for 3 to 30 min to determine uptake; or with 2 × 10^-8 ^M ciclesonide for 1 h, followed by incubation with drug-free buffer for 3, 6, and 24 h to analyze *in vitro *metabolism. High performance liquid chromatography with tandem mass spectrometry was used to measure the concentrations of both corticosteroids and metabolites.

**Results:**

At all time points the mean intracellular concentration was higher for ciclesonide when compared with fluticasone propionate. Activation of ciclesonide to desisobutyryl-ciclesonide (des-CIC) was confirmed and conjugates of des-CIC with fatty acids were detected. The intracellular concentration of ciclesonide decreased over time, whereas the concentration of des-CIC remained relatively stable: 2.27 to 3.19 pmol/dish between 3 and 24 h. The concentration of des-CIC fatty acid conjugates increased over time, with des-CIC-oleate being the main metabolite.

**Conclusion:**

Uptake of ciclesonide into A549 cells was more efficient than that of the less lipophilic fluticasone propionate. Intracellular concentrations of the pharmacologically active metabolite des-CIC were maintained for up to 24 h. The local anti-inflammatory activity of ciclesonide in the lung may be prolonged by the slow release of active drug from the depot of fatty acid esters.

## Background

Modern inhaled corticosteroids are currently the most effective drugs used in long-term asthma therapy, combining potent anti-inflammatory activity with fast metabolic inactivation [1,2]. Inhaled corticosteroids can reduce bronchial hyperresponsiveness, asthma symptoms, frequency of asthma exacerbations, and improve lung function and quality of life [3]. On the other hand, long-term treatment with inhaled corticosteroids can be associated with systemic adverse effects, such as cortisol suppression, formation of cataracts, decreased bone density, and growth suppression in children [3].

The efficacy of an inhaled corticosteroid and its potential to cause systemic adverse effects are based on its pharmacodynamic and pharmacokinetic properties [3-5]. These properties are determined by the reversible binding of the drug to the glucocorticoid receptor, which is present in almost all cell types [3]. Thus, local therapeutic benefits as well as unwanted adverse effects are mediated by the same receptor [6].

Ciclesonide is a new generation inhaled corticosteroid for the treatment of asthma and allergic rhinitis [7-9]. In contrast to other inhaled corticosteroids that bind directly to the glucocorticoid receptor, e.g. fluticasone propionate, ciclesonide is a prodrug with almost no receptor binding affinity. Airway esterases convert ciclesonide to its pharmacologically active metabolite desisobutyryl-ciclesonide (des-CIC), which has a 100-fold higher binding affinity for the glucocorticoid receptor than its parent compound [7,10]. The receptor binding affinity varies among inhaled corticosteroids and is expressed as the binding affinity relative to dexamethasone with an affinity of 100 [4]. Des-CIC and fluticasone propionate are among the most potent corticosteroids with binding affinities relative to dexamethasone of 1200 and 1800, respectively [11].

**Figure 1:**
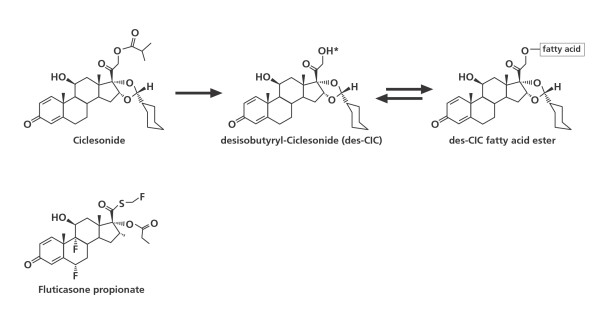
**Chemical structures of the parent compound ciclesonide, its active metabolite desisobutyryl-ciclesonide (des-CIC), des-CIC fatty acid conjugates, and fluticasone propionate**. * Hydroxyl group of des-CIC at position C-21.

Before a corticosteroid is able to bind to its receptor, it has to pass through the phospholipid bilayer of the cell membrane. The efficacy of an inhaled corticosteroid therefore also depends on its efficient uptake into the cell. Several features of the drug, such as the size of the molecule, charge, and lipophilicity play a role in its ability to cross this barrier. High lipophilicity may also enhance receptor binding affinity and this property correlates highly with the volume of distribution and pulmonary retention time of the drug [12,13]. Prolonging the time that a drug is available in the lung may enhance its anti-inflammatory activity. The conjugation of a corticosteroid with highly lipophilic fatty acids in the pulmonary tissue is a mechanism by which the retention time of a drug is increased [14]. The ester bond between the corticosteroid and the fatty acid is formed via a hydroxyl group at position C-21 [15]. Des-CIC, but not fluticasone propionate, has the required group at position C-21 (Figure 1). Conjugates of des-CIC with oleic and palmitic acid have been found *in vitro *and *in vivo *in rat and human lung tissues [16-18]. The formation of these pharmacologically inactive des-CIC fatty acid esters is a reversible process [17].

The objective of this study was to investigate the uptake of ciclesonide in human alveolar typeII epithelial cells, using the human-lung derived carcinoma A549 cell line as an established *in vitro *model for studying drug metabolism and delivery in the lung epithelium [19]. Ciclesonide uptake was compared with the uptake of fluticasone propionate, a potent and widely used inhaled corticosteroid [20]. In addition, the *in vitro *metabolism of ciclesonide in A549 cells was examined, to evaluate the formation of the pharmacologically active metabolite des-CIC and its conjugation with fatty acids in human lung epithelial cells.

## Results

### Uptake of ciclesonide and fluticasone propionate in A549 cells

**Figure 2:**
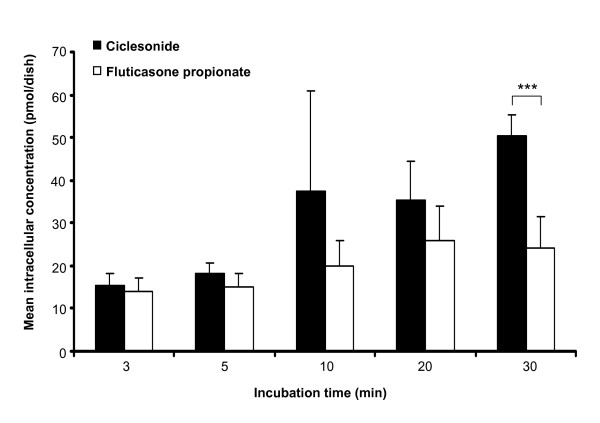
**Intracellular concentrations of ciclesonide and fluticasone propionate in A549 cells during incubation with 2 × 10-8 M ciclesonide or fluticasone propionate**. Concentrations of ciclesonide represent the sum of the intracellular concentrations of ciclesonide, des-CIC, and fatty acid esters of des-CIC. Results represent the mean ± standard deviation from 5 dishes per time point. *** p < 0.001 for ciclesonide *versus *fluticasone propionate (Aspin-Welch t-test).

The mean intracellular concentrations of ciclesonide and fluticasone propionate determined by LC/MS/MS analysis after 3, 5, 10, 20, and 30 min of incubation are shown in Table 1 and Figure 2. After 30 min, most of the ciclesonide was not metabolized. The parent compound accounted for 96% of the total amount of ciclesonide (parent compound plus metabolites). At this time point, the total concentration of ciclesonide in A549 cells was 2.1-fold higher than the concentration of fluticasone propionate; the difference was statistically significant (p < 0.001). At all other time points, the concentration of ciclesonide was numerically higher when compared with fluticasone propionate (1.1- to 1.9-fold).

**Table 1:** Intracellular concentrations of ciclesonide, des-CIC, and des-CIC-oleate during incubation with 2 × 10^-8 ^M ciclesonide

Incubation time (min)	Mean concentration ± SD (pmol/dish)^a)^		
	
	Ciclesonide	des-CIC	des-CIC-oleate^b)^
3	15.04 ± 2.48	0.41 ± 0.05	0.00 ± 0.00
5	17.86 ± 2.30	0.33 ± 0.19	0.00 ± 0.00
10	36.63 ± 23.48	0.67 ± 0.13	0.02 ± 0.05
20	34.22 ± 9.15	0.97 ± 0.14	0.12 ± 0.07
30	48.21 ± 5.19	1.72 ± 0.14	0.40 ± 0.06

^a) ^5 dishes per time point

^b) ^Conjugates of des-CIC with palmitic acid were almost undetectable.

### *In vitro *metabolism of ciclesonide in A549 cells

**Figure 3:**
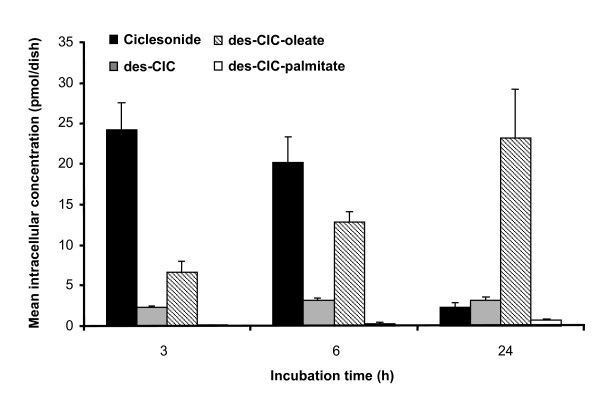
**Intracellular concentrations of ciclesonide, des-CIC, des-CIC-oleate, and des-CIC-palmitate in A549 cells during 24 h of incubation with drug-free buffer**. Results represent the mean ± standard deviation from 5 dishes per time point.

Because the majority of ciclesonide (96%) was not metabolized during 30 min of incubation, we investigated the metabolism of ciclesonide during a longer period of time. The mean intracellular concentrations of the parent compound ciclesonide, its active metabolite des-CIC and fatty acid conjugates of des-CIC after 3, 6, and 24 h of drug-free incubation are shown in Figure 3. Ciclesonide concentrations decreased over time from 24.17 ± 3.29 pmol/dish after 3 h to approximately 1/10^th ^of that level after 24 h (2.31 ± 0.55 pmol/dish). The mean intracellular concentrations of des-CIC remained about constant during the 24-h incubation period, with values of 2.27 ± 0.25, 3.10 ± 0.38, and 3.19 ± 0.38 pmol/dish after 3, 6 and 24 h of incubation. Lipid conjugates of des-CIC with oleic acid and palmitic acid were detected. At all time points, the main metabolite of ciclesonide was des-CIC-oleate. The concentrations of the des-CIC fatty acid conjugates in the A549 cells increased over time. Des-CIC-oleate concentrations increased from 6.61 ± 1.25 pmol/dish after 3 h to 23.20 ± 6.03 pmol/dish after 24 h of incubation. Des-CIC-palmitate concentrations were much lower, but showed an increase from 0.08 ± 0.08 pmol/dish to 0.74 ± 0.15 pmol/dish measured after 3 and 24 h, respectively.

## Discussion

In asthma therapy, alveolar epithelial cells are one of the most important targets for inhaled corticosteroids, because these cells are involved in the secretion of many pro-inflammatory proteins [21,22]. Thus, the efficient uptake of an inhaled corticosteroid into these cells is a prerequisite for the exertion of its anti-inflammatory activity. The human epithelial lung adenocarcinoma cell line A549 provides a useful model for drug transport and metabolic processes in epithelial type II cells [19]. In this study we evaluated the uptake efficiency of the two corticosteroids ciclesonide and fluticasone propionate into A549 cells and investigated the metabolism of ciclesonide in this cell type.

After incubation, significantly higher concentrations of ciclesonide than fluticasone propionate were detected in A549 cells, suggesting a more efficient uptake of ciclesonide into the cells (p < 0.001). These observations might be correlated with the lipophilicity of the compounds. High lipophilicity facilitates the passage of the drug through the phospholipid bilayer of the cell. The logD (logarithm of the effective partition coefficient for dissociative systems in octanol-water) values at pH 7.4 are 5.0 for ciclesonide and 4.1 for fluticasone propionate, indicating that ciclesonide has a 7.9-fold higher lipophilicity compared with fluticasone propionate.

Binding of drugs to plasma proteins is another factor that may affect the uptake of drugs into cells. In the systemic circulation, 99% of ciclesonide and 90% of fluticasone propionate are protein-bound [11], leading to the assumption that *in vivo *more unbound fluticasone propionate is available for uptake compared with ciclesonide. However, the protein content of the incubation media in these experiments was 0.1% BSA, which is lower than the protein content in the systemic circulation. Furthermore, the exact protein concentration *in vivo *on the surface of alveolar cells is not known. Therefore, it is not certain whether the difference in protein binding between the two corticosteroids might have an influence on the results of this study.

After the uptake into the A549 cells, ciclesonide is converted by airway esterases to its pharmacologically active metabolite des-CIC, which is then conjugated with fatty acids. During the 24-h drug-free incubation period, the intracellular levels of des-CIC remained stable, while ciclesonide concentrations decreased to trace amounts at 24 h and levels of des-CIC fatty acid conjugates increased over time. Des-CIC-oleate was the major fatty acid conjugate, and after 24 h of drug-free incubation, the major compound detected. Similar results were reported previously *in vitro *for precision-cut human lung slices and for human lung tissue after *in vivo *inhalation of ciclesonide [18,23]. The total amount of intracellular ciclesonide (sum of ciclesonide, des-CIC, and des-CIC-oleate) remained stable over the 24 h of drug-free incubation. In a previous study using similar experimental conditions, the anti-inflammatory potency of des-CIC in A549 cells was demonstrated by the inhibition of cytokine production [24].

## Conclusion

Overall, this study demonstrated that ciclesonide is effectively taken up into human alveolar type II epithelial (A549) cells and metabolized to its pharmacologically active metabolite des-CIC and fatty acid conjugates of des-CIC. Levels of des-CIC remained constant over a 24 h period, possibly because des-CIC may be formed from either ciclesonide or des-CIC-oleate. Fatty acid esters are much more lipophilic compared with the parent compound ciclesonide [16]. These inactive esters remain in the cells, where they reconvert to the active metabolite, thus prolonging the exposure time and local anti-inflammatory activity of ciclesonide in the lung. The slow-release pool of active drug may contribute to the proven efficacy of once-daily inhaled ciclesonide in asthma therapy, as was shown in several clinical studies [25-30].

## Methods

### Cells and materials

Human alveolar type II epithelial cells (A549) were obtained from Dainippon Pharmaceutical Co., Ltd. (Osaka. Japan). Ciclesonide, des-CIC, des-CIC-oleate, des-CIC-palmitate, deuterium-labeled des-CIC, and fluticasone propionate were supplied by ALTANA Pharma AG (Konstanz, Germany). Dulbecco's Modified Eagle Medium (DMEM), Dulbecco's phosphate-buffered saline (PBS), fetal bovine serum (FBS), and antibiotics were purchased from Invitrogen/GIBCO (Tokyo, Japan). Bovine serum albumin (BSA), ammonium acetate, and HPLC-grade ethanol (99.5%) were from Wako Pure Chemicals Industries, Ltd. (Osaka, Japan). Ethylenediamine-N, N, N', N'-tetraacetic acid tetrasodium salt (EDTA-4Na) was supplied by Dojin Laboratories, Inc. (Kumamoto, Japan). Acetonitrile was from Fisher Scientific Japan, Co. Ltd. (Tokyo, Japan), and trypsin/EDTA and trypsin neutralizing solution from Sanko Junyaku Co., Ltd. (Tokyo, Japan).

### Cell culture conditions

A549 cells were grown in DMEM supplemented with 10% (v/v) FBS at 37°C and saturated humidity (5% CO_2_, 95% air) in a CO_2 _incubator (Wakenyaku Co., Ltd., Kyoto, Japan) using 10 cm culture dishes. When confluence was reached, medium was replaced by DMEM/0.1% (w/v) BSA and cells were cultured for 24 h.

### Uptake of corticosteroids

After initial cultivation, A549 cells were washed twice with 10 ml/dish sterile PBS. Stock solutions of ciclesonide and fluticasone propionate were prepared using HPLC-grade ethanol and diluted 1000-fold with assay medium to generate the initial substrate concentration. The cells were incubated in DMEM/0.1%(w/v) BSA (5 ml/dish) with 2 × 10^-8 ^M ciclesonide or fluticasone propionate for 3, 5, 10, 20, or 30 min. At the end of the incubation period, the medium was removed by aspiration, dishes were washed five times with ice-chilled PBS (10 ml/dish) and incubated with 5 mM EDTA-4Na/PBS for further 5 minutes in the CO_2 _incubator. The cells were detached from the dishes and harvested. Each dish was washed five times with ice-chilled PBS. The cell pellets obtained by centrifugation (Kubota 8700: 1500 rpm, 5 min, 4°C) were frozen in liquid nitrogen and kept at -80°C until further use.

### Metabolism of ciclesonide

After initial cultivation, A549 cells were washed five times with 10 ml/dish sterile PBS. An ethanolic stock solution of ciclesonide was diluted 1000-fold with assay medium to generate the initial ciclesonide concentration The cells were incubated for 1 h in DMEM/0.1% (w/v) BSA (5 ml/dish) containing 2 × 10^-8 ^M ciclesonide as described above in a CO_2 _incubator. At the end of the incubation period, dishes were washed five times with PBS and incubated for 3, 6, or 24 h at the same conditions in 5 ml/dish fresh medium (DMEM/0.1% (w/v) BSA). At each indicated time point, the medium was removed by aspiration, 5 mM EDTA-4Na/PBS added and dishes were incubated for further 5 min. The cells were detached from the dishes and harvested. Each dish was washed five times with ice-chilled PBS. The cell pellets obtained by centrifugation were frozen in liquid nitrogen and kept at -80°C until further use.

### Preparation of cell extracts

Cell pellets were homogenized in 0.8 ml ethanol using an ultrasonic homogenizer (Sonifier 250, Branson Ultrasonics Corporation, Danbury, Conn, USA). After centrifugation for 15 min at 4°C, aliquots of the supernatant were stored at -20°C until further use.

### Liquid chromatography and tandem mass spectrometry (LC/MS/MS)

Ciclesonide, des-CIC, des-CIC fatty acid conjugates, and fluticasone propionate were separated by high performance liquid chromatography (HPLC; Agilent HP1100, Agilent Technologies, Tokyo, Japan) using a 5μm Hypersil^® ^Phenyl 2 column (50 × 4.6 mm; Thermo Electron, K.K., Yokohama, Japan) The mobile phase consisted of 25% (v/v) acetonitrile/purified water/1 mM ammonium acetate and 95% (v/v) acetonitrile/purified water/1 mM ammonium acetate at a flow rate of 1.0 ml/min for a total run time of 7 min. As an internal standard, an analogue of des-CIC carrying a deuterium label at the cyclohexyl group was used. The analytes were detected by a tandem mass spectrometry system (API3000, Applied Biosystems, Tokyo, Japan) with a turbo ion spray source and negative multiple reaction monitoring scan mode.

## Data analysis

The concentrations of ciclesonide, fluticasone propionate, and metabolites in the assay samples were determined by comparing calibration curves using the Analyst 1.1 data processing program. The calibration curves were drawn from the primary regression line determined by the least-squares method (weighting factor: 1/x). The lower limits of quantification (LLOQ), determined from the calibration curves, were defined as 0.10 ng/ml for ciclesonide and des-CIC-oleate, 0.20 ng/ml for des-CIC and des-CIC-palmitate, and 0.40 ng/ml for fluticasone propionate.

Differences in concentrations between samples treated with either ciclesonide or fluticasone dipropionate were determined by using the Aspin Welch t-test. The concentrations are given as mean ± standard deviation.

## Competing interests

The author(s) declares that there are no competing interests.

## Authors' contributions

TN, RN, AK, and YK were involved in planning the experiments. TN and AK performed the experiments and analyzed the data. RN and NM drafted the manuscript, which was read and approved by all authors.
